# Association of the Resistin Gene Promoter Region Polymorphism with Kawasaki Disease in Chinese Children

**DOI:** 10.1155/2012/356362

**Published:** 2012-04-08

**Authors:** Ruixi Liu, Bo He, Fang Gao, Qian Liu, Qijian Yi

**Affiliations:** Department of Cardiovascular Medicine, Children's Hospital of Chongqing Medical University and Ministry of Education Key Laboratory of Child Development and Disorders and Key Laboratory of Pediatrics in Chongqing, CSTC2009CA5002, Chongqing International Science and Technology and Cooperation Center for Child Development and Disorders, Yuzhong District, Chongqing 400014, China

## Abstract

*Objectives*. The −420 C > G polymorphism located in the resistin gene (RETN) promoter has recently been suggested to play a potential role in proinflammatory conditions and cardiovascular disease. This study investigated the association of the RETN promoter polymorphism with Kawasaki disease (KD) and its clinical parameters in Chinese children. *Methods*. We compared patients with complete KD to incomplete KD children. Genotyping of the RETN promoter polymorphism was performed using MassARRAY system, and serum resistin levels were estimated using the sandwich enzyme immunoassay method. *Results*. There was no significant difference in RETN (−420 C > G) genotypes between KD and control groups. However, the frequency of the G allele was higher in iKD patients than in cKD children due to a significantly increased frequency of the GG genotypes. Serum levels of resistin were significantly higher in KD patients than in controls regardless of the presence of coronary artery lesions (CALs). *Conclusion*. The present findings suggest that while resistin may play a role in the pathogenesis of KD, there is no apparent association between CAL and the RETN (−420 C > G) gene polymorphism in KD children. However, the diagnosis of iKD is challenging but can be supported by the presence of the G allele and the GG genotypes.

## 1. Introduction

Kawasaki is an acute systemic vasculitis that predominantly affects infants and young children. The most serious complication of KD is the occurrence of coronary artery lesions (CAL), including myocardial infarction, coronary artery fistula formation [1], coronary artery dilatation, or coronary artery aneurysm [2]. Some evidence suggests that the excess production of inflammatory mediators is involved in the pathology of KD [3].

Although more than 40 years have passed since the first description of KD [4], no specific laboratory markers have been identified to date, and the diagnosis consequently rests on the clinical findings. Some patients fail to meet all the criteria [2] for KD but are nevertheless at risk for the development of coronary artery disease. These incomplete forms of KD (iKD) are being increasingly reported, although no clear definition has been developed [2, 5]. In patients with iKD, the diagnosis is challenging and therapeutic decisions are difficult.

Resistin, a novel adipocyte-derived peptide, belongs to a family of cysteine-rich secretory proteins [6]. In humans, the peptide is expressed in monocytes and macrophages [7], and it is considered to be involved in the pathogenesis of inflammation [8], coronary artery calcification [9], atherosclerosis, and acute coronary syndrome [10].

RETN, the gene coding for human resistin, is located on chromosome 19p13.3 [6]. To date, several single-nucleotide polymorphisms in the RETN gene have been described [11, 12]. One of the most frequently studied polymorphisms, RETN −420 C > *G* (rs1862513), was reported to be associated with the regulation of RETN gene expression and serum resistin level [13–15]. Several studies have also associated the RETN −420 C > *G* polymorphism with arteriosclerosis, coronary artery disease [16] and cerebrovascular disease [17]. To our knowledge, no data have been published on the association of the RETN −420 C > *G* polymorphism with the risk of KD.

In this study, we hypothesized that the genetic polymorphisms of RETN −420 C > *G* was associated with KD susceptibility and/or CAL formation. We investigated the association of RETN −420 C > *G* polymorphisms in the susceptibility of KD (cKD and iKD) patients and control subjects as well as KD patients with and without CAL formation.

## 2. Subjects and Methods

### 2.1. Subjects and Data Collection

We enrolled patients with KD from the Children's Hospital of Chongqing Medicine University, Chongqing, China. The study group included 91 patients (58 males and 33 females, 3.31 ± 2.74 year old); all of whom met the criteria proposed by the Japanese Kawasaki Disease Research Committee [18]. According to the criteria of KD, there were 91 identified patients, classified as cKD (*n* = 57) or iKD (*n* = 34), cKD was defined as a fever and at least four of the five principal criteria [2], and iKD only meet as fever persisting at least 5 days and one to three principal criteria. These patients were treated with oral aspirin and 1 or 2 g/kg intravenous immune globulin after admission. Sixty-eight patients (74.7%) responded to intravenous immune globulin infusion. In addition, 115 sex-age-matched healthy blood subjects were used as healthy controls. The study protocol was approved by the Ethics Committee of the Chongqing Medicine University, and written informed consents were obtained from the parents of all subjects.

Echocardiography was obtained within 2 weeks of the onset or before intravenous immunoglobulin administration. CALs were defined as coronary vessel internal diameter ≥2SDs above the mean for age adjusted for body surface area (BSA) [19]. Patients were divided into two groups according to the presence of CAL: 35 patients with CALs and 56 patients without CALs.

Laboratory data were obtained for each child, including white blood cell (WBC) counts, red blood cell (RBC) counts, hemoglobin (HB), platelet count, alanine aminotransferase (ALT) level, aspartate aminotransferase (AST) level, C-reactive protein (CRP), and erythrocyte sedimentation rate (ESR). All blood samples were drawn before IVIG therapy in the KD patient group.

### 2.2. Sample Collection and Processing

Venous blood samples were obtained from patients and healthy subjects. For biochemical analysis, blood samples were allowed to clot at room temperature, then immediately centrifuged to separate serum, and kept at −80°C. For molecular study, blood samples were collected in ethylenediaminetetraacetic acid (EDTA) tubes to prevent the coagulation of blood samples. Genomic DNA was extracted from whole blood using standard phenol-chloroform extraction technique.

### 2.3. Measurement of Serum Resistin Levels

Resistin levels from the serum samples were determined using commercial immunoassay kit according to the manufacturer's instructions (R&D Systems, Minneapolis, MN, USA), as described previously [20]. Resistin standards were run on microTest plates, and the antigen concentration (ng/mL) was determined from the standard curve using the AMP Diagnostics ELISA reader (Austria).

### 2.4. Genotyping of the RETN −420 C/G Polymorphism

SNP genotyping was done using MassARRAY system (Sequenom) by means of matrix-assisted laser desorption ionisation-time of flight mass spectrometry method (MALDI-TOF) according to the manufacturer's instructions (Shanghai Benegene Biotechnology Co., Ltd). Genotype calling was performed in real time with MassARRAY RT software version 3.0.0.4 and analyzed using the MassARRAY Typer software version 3.4 (Sequenom).

### 2.5. Statistical Analysis

Clinical phenotypes, including WBC counts, platelet counts, RBC counts, HB levels, platelet count, ALT level, AST level, CRP, and ESR, were each analyzed as a quantitative trait. All values in this study are described as mean ± standard deviation (SD). Statistical significance of the differences between the continuous variables was evaluated by Student's *t*-test or the Mann-Whitney test. Analysis of variance (ANOVA) followed by Scheffe's test was used for analysis of the serum levels of each genotype. The probability values presented are based on two-sided tests. Statistical analysis was performed using the program SPSS for Windows program version 12.0 (SPSS, Chicago, IL, USA). A two-sided *P* value less than 0.05 was considered statistically significant.

## 3. Results

### 3.1. Clinical Characteristics of Study Subjects

As shown in Table 1, platelet count, WBC counts, RBC counts, ALT level, AST level, CRP, and ESR were higher in KD patients compared to controls (*P* < 0.001), whereas hemoglobin and HB levels were significantly lower in KD patients compared to controls (*P* < 0.05).

The comparison of parameters between patients with CALs and patients without CALs was presented in Table 2. There were no significant differences between two groups in age, ALT, AST, CRP, platelet count, WBC counts, and ESR levels but HB levels (*P* < 0.05).

The characteristics of cKD patients and iKD groups are presented in Table 3. Compared with cKD groups significant lower CRP (*P* < 0.001) and higher HB levels and platelet count (*P* all <0.05) were noted in patients with iKD. 

### 3.2. Allele Frequencies of the RETN (−420 C/G) Polymorphism in KD and Control Subjects

Ninety-one children with KD and 115 healthy children were genotyped at the RETN (−420 C/G) polymorphism, and allele frequencies were examined. The genomic frequencies of the RETN (−420 C/G) polymorphism in children with KD were 17.6% (16/91) for CC, 46.2% (42/91) for CG, and 36.3% (33/91) for GG. In controls, these frequencies were 12.2% (14/115) for CC, 45.2% (52/115) for CG, and 42.6% (49/115) for GG. The genomic and allelic frequencies at this polymorphism did not significantly differ between children with KD and control subjects (Table 4).

### 3.3. Allele Frequencies of the RETN (−420 C/G) Polymorphism in KD Children with and without Coronary Lesions

The frequency of the combined CG + CC genotype was 67.9% (38/56) in KD patients without CALs and 74.3% (26/35) in KD patients with CALs. There was no significant difference in genotype between KD children with and without CALs (Table 5). These results suggest that the RETN (−420 C/G) polymorphism may not be associated with the development of CALs in Chinese children with KD.

### 3.4. Allele Frequencies of the RETN (−420 C/G) Polymorphism in Patients with cKD and Groups with iKD

 The genomic frequencies in children with cKD were 18.8% (11/57) for CC, 60.4% (34/57) for CG, and 20.8% (12/57) for GG. In children with iKD, these frequencies were 11.8% (4/34) for CC, 35.3% (12/34) for CG, and 52.9% (18/34) for GG. Statistical analysis revealed that significantly more iKD than cKD patients were homozygous for the G allele (aOR 2.302, 95% CI 0.995–5.38; Table 6).

### 3.5. Allele Frequencies of the RETN (−420 C/G) Polymorphism in Responders and Nonresponders to Intravenous Immune Globulin Therapy

 There was no significant difference in genomic frequencies and genotype between responders and nonresponders (Table 7).

### 3.6. Association between the RETN (−420 C/G) Polymorphism and Clinical Parameters in Children with KD

There were no significant differences in WBC count, hemoglobin level, platelet count, CRP, ALT, or AST among KD children with genotypes GG, CG, and CC (Table 8).

### 3.7. Serum Resistin Concentration

**Figure 1:**
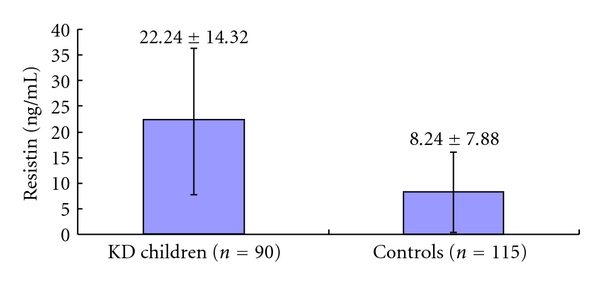
Serum resistin levels in children with the Kawasaki disease (KD) and control subjects.

**Figure 2:**
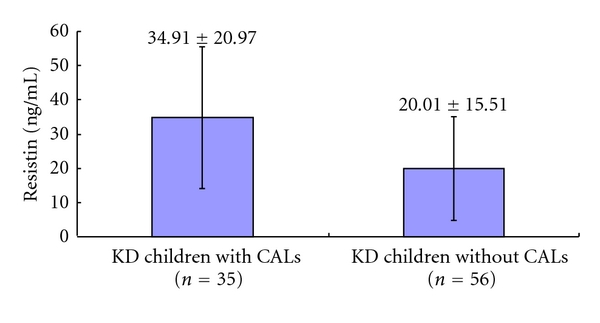
Serum resistin levels in the Kawasaki disease (KD) children with and without CALs.

**Figure 3:**
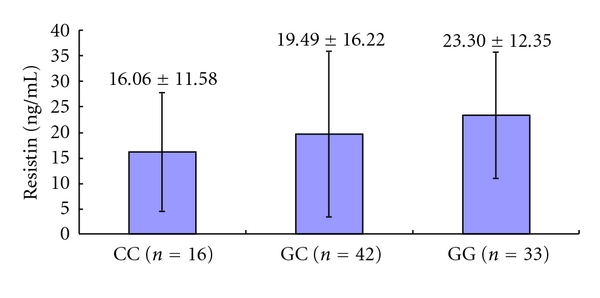
Serum resistin levels in Kawasaki disease (KD) children with CC, GC, and GG genotypes.

Serum resistin levels were significantly higher in KD patients than control subjects (20.24 ± 14.32 ng/mL versus 8.24 ± 7.88 ng/mL, *P* < 0.001; Figure 1). However, the level was not significantly higher in KD patients with CALs compared to KD patients without CALs (25.69 ± 17.58 ng/mL versus 20.84 ± 13.74 ng/mL, *P* = 0.25; Figure 2). Serum resistin levels were higher in the GG group compared to the CG and CC groups among KD patients, but the difference was not significant (Figure 3, *P* > 0.05).

## 4. Discussion

To our knowledge, this is the first study to examine the association between the RETN −420 C > *G* polymorphism and the risk of KD. We herein investigated whether the RETN −420 C > *G* promoter polymorphism could be associated with the development of CALs in KD patients. Among our study population, we found no significant difference in RETN −420 C > *G* genotypes between KD and control subjects. In addition, there was no association between the RETN −420 C > *G* gene polymorphism and development of CALs in the enrolled KD patients. However, our analysis identified a statistically significant association between the RETN −420 C to G mutation and the increased risk of iKD. These findings suggest that the tested RETN −420 C > *G* polymorphism might not be associated with the development of KD and CALs in Chinese children with KD. However, the diagnosis of iKD is challenging but can be supported by the presence of RETN (−420 C > G) GG genotype or G allele.

In our control group, the −420 G allele frequency was observed at 65.2%, which was higher than that reported in the study by Xu et al. (>31% in Hong Kong, China) [21]. This inconsistency might reflect the selection bias. The RETN −420 C > *G* polymorphism is located in the promoter region of the RETN gene. To date, many studies have estimated the associations between the RETN −420 C > *G* polymorphism and coronary artery disease, although the results are inconsistent. Kunnari et al. investigated the associations between the RETN −420 C > *G* polymorphism and cerebrovascular disease in Finnish type 2 diabetes patients [17]. Their study demonstrated that the GG homozygote was associated with a higher frequency of cerebrovascular disease, suggesting a potential role for the RETN −420 C > *G* polymorphism in atherogenesis. In recent paper Tang and colleagues discuss an association of the G allele with cardiovascular disease in a Chinese population [16]. In contrast to Tang et al., Michael et al. could not find that this polymorphism is a risk factor for cardiovascular disease [22]. In the present study, we conducted a hospital-based case-control study to examine the association between the RETN −420 C > *G* polymorphism and the risk of KD. Among our study population, we found no significant difference in RETN −420 C > *G* genotypes between KD and control subjects. In addition, there was no association between the RETN −420 C > *G* gene polymorphism and development of CALs in the enrolled KD patients. However, we found increased risk of iKD in subjects with RETN (−420 C > G) GG genotype compared with carriers of other genotypes (CC and CG). In recent years, patients who did not fulfill all the clinical criteria of KD have been described. Moreover, iKD group is associated with an increased risk of coronary aneurysm [23, 24]. Given the diagnostic difficulties, the diagnostic criterion of incomplete KD cases remains unclearly defined. Alternatively, the presence of coronary artery aneurysms may be the only definitive means to diagnose iKD. Fortuitously, our study found that significantly more iKD than cKD patients were homozygous for the G allele. Therefore, the diagnosis of iKD is challenging but can be supported by the presence of RETN (−420 C > G) G allele or GG genotype.

It has been reported that the RETN −420 C > G variant could gain the ability to bind the Sp1/3 transcription factor, which might markedly enhance RETN gene promoter activity and increase transcription of the RETN gene [15]. Previous studies have also demonstrated that the RETN −420 G allele was associated with higher expression of resistin mRNA and resistin levels in humans [13, 14]. Moreover, the expression of resistin mRNA in monocytes was reported to be highest in subjects with the RETN −420 GG genotype, followed by CG and CC [25]. We observed that serum resistin levels were higher in the CG + GG group compared to the CC group in Chinese children with KD, but this difference did not reach statistical significance. This association is also supported by a study reported by Menzaghi et al. in an analysis of Caucasian subjects [26]. Most recently, Hivert et al. also reported that SNP-420 was not associated with circulating resistin in Caucasian subjects [27]. In their meta-analysis, which included Caucasians, Japanese, and Korean subjects, SNP-420 was found to be associated with plasma resistin with significant heterogeneity among the groups studied, suggesting that ethnic differences may be a factor [27]. The present study is limited by the relatively small study population and the possibility of linkage disequilibrium with an unknown risk allele in or near the RETN gene. Further large-scale studies will be necessary to examine associations between RETN gene polymorphisms and serum resistin levels in KD patients with and without CAL. In addition, it is unknown whether or not the polymorphism examined herein plays a functional role in resistin expression.

Some studies have also shown correlations between KD with CALs and inflammatory parameters such as CRP [28, 29]. Furthermore, interleukin (IL)-6 and tumor necrosis factor-*α* levels were also significantly higher in KD patients with coronary aneurysm compared to those without coronary aneurysm [30]. In recent years, the role of resistin in the pathogenesis of cardiovascular and inflammatory disease has become of increasing interest. Resistin has been shown to be involved in the process of inflammation, which is known to play a crucial role in the development of coronary artery disease [8]. In previous literature, it is shown that resistin is induced by lipopolysaccharides (LPSs) and TNF-alpha in macrophages in vitro and is markedly increased in leukocytes and plasma in vivo during endotoxaemia [31]. It is reported to strongly upregulate the expression of proinflammatory cytokines including interleukin (IL)-6 and tumour necrosis factor (TNF)-*α* in human and is associated with many inflammatory markers including C-reactive protein and intercellular cell-adhesion molecule (ICAM)-1 [9]. Resistin also competes with LPS to bind TLR-4 (which is a toll-like receptor on mononuclear cells) so it acts like a proinflammatory cytokine [32].Therefore, it was reasonable to assume that serum resistin levels were significantly higher in KD patients than control subjects. Our finding of the increased levels of resistin from KD patients is in agreement with the previous research in Japanese patients with KD [33]. In a recent study, the results suggest that high serum resistin level may be a predictor of nonresponsiveness to intravenous immune globulin therapy [34]. However, there were no obvious differences in the genomic frequencies and genotype between responders and nonresponders Therefore, more studies are needed to confirm the hypothesis that the association between resistin concentration and the nonresponders.

We also estimated the impact of the RETN −420 C > *G* polymorphism on the clinical parameters in KD patients. Our data showed that there were no significant differences between the genotypes with respect to WBC count, hemoglobin level, platelet count, CRP, ALT, and AST in KD patients. Several previous studies have focused on the associations between the RETN −420 C > *G* polymorphism and CRP levels, and no consistent results have been reported. Tang and associates have demonstrated that the RETN −420 C > *G* polymorphism leads to increased CRP concentrations in patients with stable CAD, suggesting a potential involvement of the protein in atherogenesis [35]. However, we did not find any significant correlations between the RETN −420 C > *G* polymorphism and CRP. The inconsistency between these findings may be due to the difference in the region origin of the study populations.

The potential limitations of our study should be noted. First, hospital-based controls were used, possibly resulting in selection bias. However, the genotype distribution of controls in our study was compatible with the Hardy-Weinberg expectations. Second, our study was conducted in a Chinese population, and the data should be extrapolated to other ethnic groups with caution. Third, the sample size of the present study was relatively small, possibly resulting in wide confidence intervals that approach unity. Larger studies are required to confirm our observations. Lastly, we did not get the data of BMI (body mass index), and BMI is an important factor which may independently effect resistin levels [31]. However, this internally consistent pilot study certainly provides valuable insights and interesting information and may serve to guide future studies in this area.

In conclusion, this study demonstrates for the first time that the RETN −420 C > *G* polymorphism is associated with the presence of iKD. Although resistin appears to play a role in KD, the RETN (−420 C/G) gene polymorphism does not appear to be associated with the development of KD or CAL in Chinese children. However, further large-scale studies will be needed to firmly establish the relationships between RETN gene polymorphisms and CAL in KD. Collectively, this work will provide important new insights into the KD pathology and may provide new targets for the design of novel diagnosis measures for iKD.

## Figures and Tables

**Table 1:** Comparison of laboratory characteristic of patients and control groups.

	KD (cKD and iKD *n* = 91)	Controls (*n* = 115)	*P*
Age at diagnosis (yr)	3.31 ± 2.74	2.10 ± 1.04	0.232
Sex (male/female)	58/33	75/40	0.557
Hemoglobin (g/L)	103.60 ± 8.86	124.02 ± 16.84	<0.001
Platelet (10^3^/uL)	427.24 ± 162.57	255.87 ± 43.49	<0.001
WBC (10^3^/uL)	13.02 ± 6.06	7.35 ± 1.40	<0.001
RBC (10^6^/mm^3^)	3.97 ± 0.49	4.43 ± 0.29	<0.001
AST (U/L)	57.05 ± 86.80	17.60 ± 6.49	<0.001
ALT (U/L)	85.19 ± 107.69	27.6 ± 6.80	<0.001
CRP (mg/dL)	47.29 ± 51.61	4.39 ± 1.30	<0.001
ESR (mm/hr)	55.97 ± 27.21	7.89 ± 3.30	<0.001
Resistin (ng/mL)	22.24 ± 14.32	8.24 ± 7.88	<0.001

Notes: KD, Kawasaki disease; yr, year; WBC, white blood cell counts; CRP, C-reactive protein; ESR, erythrocyte sedimentation rate; red blood cells counts (RBC); alanine aminotransferase (ALT) level; aspartate aminotransferase (AST). *P *value is for comparison between control and patients.

**Table 2:** Relation between clinical parameters in KD patients and development of CALs.

	KD with CALs (*n* = 35)	KD without CALs (*n* = 56)	*P*
Age at diagnosis (yr)	2.99 ± 2.79	3.52 ± 2.31	0.463
Hemoglobin (g/L)	101.31 ± 10.25	107.16 ± 10.06	0.048
Platelet (10^3^/uL)	418.60 ± 183.58	419.05 ± 188.85	0.993
WBC (10^3^/uL)	14.50 ± 5.71	11.12 ± 7.30	0.066
RBC (10^6^/mm^3^)	3.99 ± 0.55	3.98 ± 0.51	0.988
AST (U/L)	53.32 ± 83.94	57.82 ± 75.58	0.853
ALT (U/L)	75.62 ± 87.06	90.23 ± 128.25	0.633
CRP (mg/dL)	45.29 ± 46.03	52.21 ± 63.16	0.646
ESR (mm/hr)	52.89 ± 30.60	49.59 ± 25.80	0.730
Resistin (ng/mL)	34.91 ± 20.97	20.01 ± 15.51	0.066

Notes: CAL: coronary artery lesion; *P* value is for comparison between patients with CALs and these without CALs.

**Table 3:** Clinical and laboratory variables at admission in the groups with complete and incomplete KD.

	Complete KD (*n* = 57)	Incomplete KD (*n* = 34)	*P*
Age at diagnosis (yr)	3.09 ± 2.40	3.82 ± 3.74	0.433
Hemoglobin (g/L)	101.49 ± 9.94	109.92 ± 10.04	0.012
Platelet (10^3^/uL)	389.88 ± 183.28	522.25 ± 149.65	0.026
WBC (10^3^/uL)	13.31 ± 6.71	18.41 ± 4.30	0.174
RBC (10^6^/mm^3^)	3.93 ± 0.52	4.19 ± 0.53	0.128
AST (U/L)	59.82 ± 88.82	35.11 ± 20.20	0.414
ALT (U/L)	91.62 ± 109.06	34.23 ± 32.25	0.136
CRP (mg/dL)	58.42 ± 54.81	13.63 ± 15.10	0.000
ESR (mm/hr)	52.92 ± 26.60	36.59 ± 10.80	0.550
Resistin (ng/mL)	17.01 ± 13.13	24.41 ± 16.38	0.155

Note: *P* value is for comparison between groups with complete and incomplete KD.

**Table 4:** RETN (−420 *C* > *G*) genotype and allele frequency in KD patients and controls.

		KD (*n* = 91) *n* (%)	Control (*n* = 115) *n* (%)	*P* value	OR (95% CI)
Allele	C	74 (0.407)	80 (0.348)		
	G	108 (0.593)	150 (0.652)	0.221	1.285 (0.860–1.919)
Genotypes	CC	16 (0.176)	14 (0.122)		
	GG	33 (0.363)	49 (0.426)		
	CG	42 (0.462)	52 (0.452)	0.462	
Genotypes	GG	33 (0.363)	49 (0.426)		
	CC + C G	58 (0.637)	66 (0.574)	0.356	0.766 (0.436–1.413)

Notes: RETN: resistin gene; OR: odds ratio; CI: confidence interval. *P* value is for comparison between control and patients.

**Table 5:** RETN (−420 *C* > *G*) genotype and allele frequency in the groups with CALs and without CALs.

		KD with CALs (*n* = 35) *n* (%)	KD without CALs (*n* = 56) *n* (%)	*P* value	OR (95% CI)
Allele	C	34 (0.486)	44 (0.393)		
	G	36 (0.514)	68 (0.607)	0.297	0.685 (0.336–1.397)
Genotypes	CC	8 (0.229)	6 (0.107)		
	GG	9 (0.257)	18 (0.514)		
	CG	18 (0.514)	32 (0.571)	0.442	
Genotypes	GG	9 (0.257)	18 (0.321)		
	CC + CG	26 (0.743)	38 (0.679)	0.575	1.368 (0.457–4.099)

Note:* P* value is for comparison between patients with CALs and these without CALs.

**Table 6:** RETN (−420 *C* > *G*) genotype and allele frequency in the groups with complete and incomplete KD.

		Complete KD (*n* = 57) *n* (%)	Incomplete KD (*n* = 34) *n* (%)	*P* value	OR (95% CI)
Allele	C	56 (0.490)	20 (0.294)		
	G	58 (0.510)	48 (0.706)	0.048	2.302 (0.995–5.328)
Genotypes	CC	11 (0.188)	4 (0.118)		
	GG	12 (0.208)	18 (0.529)		
	CG	34 (0.604)	12 (0.353)	0.044	
Genotypes	GG	12 (0.208)	18 (0.529)		
	CC + CG	45 (0.792)	16 (0.471)	0.012	0.234 (0.072–0.761)

Note: *P* value is for comparison between groups with complete and incomplete KD.

**Table 7:** RETN (−420 *C* > *G*) genotype and allele frequency in the groups with responders and nonresponders.

		Responders (*n* = 68) *n* (%)	Nonresponders (*n* = 23) *n* (%)	*P* value	OR (95% CI)
Allele	C	52 (0.388)	16 (0.348)	0.752	1.224 (0.349–4.293)
	G	82 (0.612)	30 (0.652)		
Genotypes	CC	8 (0.134)	0 (0.000)	0.504	
	GG	23 (0.333)	7 (0.286)		
	CG	36 (0.524)	16 (0.714)		
Genotypes	GG	23 (0.343)	7 (0.304)	0.815	1.25 (0.192–8.144)
	CC + CG	44 (0.657)	16 (0.696)		

Note: *P* value is for comparison between groups with responders and nonresponders

**Table 8:** Association between RETN (−420 *C* > *G*) alleles and clinical and laboratory data obtained from children with the Kawasaki disease.

Clinical and laboratory data	GG	CC + CG	*P*
Hemoglobin (g/L)	107.5 ± 7.43	110.13 ± 2.34	0.240
Platelet (10^3^/uL)	329.43 ± 74.28	461.77 ± 200.29	0.102
WBC (10^3^/uL)	13.04 ± 5.18	13.78 ± 6.38	0.771
RBC (10^6^/mm^3^)	3.89 ± 0.39	3.93 ± 0.41	0.79
AST (U/L)	42.16 ± 22.77	68.70 ± 103.77	0.545
ALT (U/L)	82.33 ± 71.54	93.00 ± 123.81	0.844
CRP (mg/dL)	45.57 ± 35.44	37.42 ± 33.15	0.585
ESR (mm/hr)	48.38 ± 27.42	53.42 ± 28.34	0.674
Resistin (ng/mL)	23.30 ± 12.35	19.49 ± 16.22	0.550

Note: *P* value is for comparison between GG genotype and CC + CG genotype in children with KD.
